# Relationship between *ABCB1* 3435TT genotype and antiepileptic drugs resistance in Epilepsy: updated systematic review and meta-analysis

**DOI:** 10.1186/s12883-017-0801-x

**Published:** 2017-02-15

**Authors:** Malek Chouchi, Wajih Kaabachi, Hedia Klaa, Kalthoum Tizaoui, Ilhem Ben-Youssef Turki, Lamia Hila

**Affiliations:** 1 Department of Genetic, Tunis El Manar University, Faculty of Medicine of Tunis, 15 Jebel Lakhdhar street, La Rabta, 1007 Tunis, Tunisia; 2 Department of Child Neurology, National Institute Mongi Ben Hmida of Neurology, UR12SP24 Abnormal Movements of Neurologic Diseases, Jebel Lakhdhar street, La Rabta, 1007 Tunis, Tunisia; 3 Division of Histology and Immunology Division, Department of Basic Sciences, Faculty of Medicine of Tunis, 15 Jebel Lakhdhar street, La Rabta, 1007 Tunis, Tunisia; 4 Department of Genetic, Faculty of Medicine of Tunis, 15 Jebel Lakhdhar street, La Rabta, 1007 Tunis, Tunisia

**Keywords:** Epilepsy, Antiepileptic drugs, Resistance, *ABCB1* C3435T polymorphism, Meta-analysis

## Abstract

**Background:**

Antiepileptic drugs (AEDs) are effective medications available for epilepsy. However, many patients do not respond to this treatment and become resistant. Genetic polymorphisms may be involved in the variation of AEDs response. Therefore, we conducted an updated systematic review and a meta-analysis to investigate the contribution of the genetic profile on epilepsy drug resistance.

**Methods:**

We proceeded to the selection of eligible studies related to the associations of polymorphisms with resistance to AEDs therapy in epilepsy, published from January 1980 until November 2016, using Pubmed and Cochrane Library databases. The association analysis was based on pooled odds ratios (ORs) and 95% confidence intervals (CIs).

**Results:**

From 640 articles, we retained 13 articles to evaluate the relationship between ATP-binding cassette sub-family C member 1 (*ABCB1*) C3435T polymorphism and AEDs responsiveness in a total of 454 epileptic AEDs-resistant cases and 282 AEDs-responsive cases. We found a significant association with an OR of 1.877, 95% CI 1.213–2.905. Subanalysis by genotype model showed a more significant association between the recessive model of *ABCB1* C3435T polymorphism (TT vs. CC) and the risk of AEDs resistance with an OR of 2.375, 95% CI 1.775–3.178 than in the dominant one (CC vs. TT) with an OR of 1.686, 95% CI 0.877–3.242.

**Conclusion:**

Our results indicate that *ABCB1* C3435T polymorphism, especially TT genotype, plays an important role in refractory epilepsy. As genetic screening of this genotype may be useful to predict AEDs response before starting the treatment, further investigations should validate the association.

## Background

Epilepsy is a chronic neurological worldwide disorder [1]. Most cases of epileptic patients respond to antiepileptic drugs (AEDs). However, about one-third of epileptic patients develop recurrent seizures, despite the efficacy of treatment at the optimal dose regimen. They are then, considered resistant to antiepileptic treatment [2]. The international league against epilepsy (ILAE) redefined refractory epilepsy in 2010 as the persistence of seizures after two adequate trials of appropriate and tolerated AEDs [3].

The exact mechanism of refractory epilepsy is not well understood. Two main hypotheses are potentially involved in the biological mechanism of AEDs resistance: transporter and target hypotheses. The transporter hypothesis supports the overexpression of drug efflux transporters at the blood–brain barrier (BBB) reducing AEDs access to the brain. The target hypothesis contends that the changes in drug intracellular target sites (receptors) result in decreased sensitivity of AEDs [4, 5]. Therefore, the two mechanisms prevent pharmacological effects of antiepileptic at cerebral sites initiating seizures. It seems that genetic polymorphisms of drug transporter and target genes have a potential impact on the resistance to treatment: they may be responsible for the mechanisms of intractable epilepsy [5–7] by changing the function of genes products [8–10] and leading to the AEDs failure [4, 11–14]. Moreover, other authors have suggested that they may involve the prognosis of newly treated epilepsy [15]. Since drug-resistant epilepsy represents a major problem in the control of seizures, the researchers focused on the genetic profile to try to better understand the pharmacoresistance for a more effective treatment.

Since drug resistance often occurs in patients with multiple AEDs, the multidrug transporter hypothesis is considered better than the target hypothesis to explain the phenomenon of AEDs resistant epilepsy. However, the two hypotheses may complement each other. Given that drug transport mechanisms are the candidate mechanisms underlying AEDs resistance [16], many studies took significantly into consideration the association between efflux transporters overexpression inducing recurrent seizures.

Bioavailability and response to medication in epilepsy are mainly influenced by atp-binding cassette (ABC) transporter superfamily. The atp-binding cassette sub-family b member 1 (*ABCB1*) and the atp-binding cassette sub-family c member 2 (*ABCC2*) also known as multidrug resistance protein 1 (*MDR1*) and multidrug resistance protein 2 (*MDR2*), located at the membrane of BBB endothelial cells, are members of the ABC superfamily. They are the most studied candidate genes in pharmacoresistant epilepsy [5]. P-glycoprotein (P-gp) was the first human ABC protein that has been discovered [17]. *ABCB1* gene encodes it and it affects a wide range of drugs distribution in target compartments [18–20]. The C3435T polymorphism is the most investigated polymorphism in the *ABCB1* gene (single nucleotide polymorphism (SNP) in exon 26) and it has received the most attention. It has been associated with the variations in the expression levels of P-gp [21]. Previous studies focusing on the association between *ABCB1* C3435T polymorphism and drug-resistant epilepsy showed discordant findings. Several studies have supported the hypothesis of this association (alleles, genotypes or haplotypes) to AEDs resistance [22–37]. However, a number of studies conducted on epileptic patients from different regions and ethnicities failed to confirm this result [38–42]. Subsequently, the opposed findings stimulated some previous meta-analyses of which the majority indicated that no association existed [43–49]. Besides, G1249A polymorphism is one of the common polymorphisms in the *ABCC2* gene (SNP in exon 10). The overexpression of the ABCC2 transporter protein reduces AEDs levels in brain tissues, which is a risk factor for pharmacoresistant epilepsy. A genotypic association between this polymorphism and responsiveness to AEDs has been suggested in Asian populations [50, 51]. However, other studies published contradictory results and they did not find any association [42, 52–56]. Furthermore, only two meta-analyses investigated its role in drug-resistant epilepsy and found that *ABCC2* G1249A polymorphism was significantly associated with the decreased risk of AED resistance [57, 58].

Among their pharmacological effects, some AEDs may block voltage-dependent sodium channels [59, 60], which stimulate the researchers to investigate the potential link between drug-resistant epilepsy and polymorphisms in channels genes like *SCN1A* gene. This gene is the most studied drug target gene in epilepsy and it exhibits an intronic polymorphism IVS5-91G > A, one of the most common polymorphisms (SNP at intron splice donor site of exon 5). It alters the proportion of human brain NaV1.1-5N (exon 5N) and NaV1.1-5A (exon 5A) proteins, but the functional impact of the splicing on NaV1.1 is unknown. The correlation between *SCN1A* IVS5-91G > A polymorphism and maximum doses of Oxcarbazepine (OXC) may have a potential effect on resistant to epilepsy. The same study found the same correlation for *ABCC2* G1249A polymorphism [61]. An additional study reported a genotypic association of *SCN1A* IVS5-91G > A polymorphism with the response to Carbamazepine (CBZ)/OXC [51, 62], and another one showed its role on pharmacoresponse to CBZ via an effect on GABAergic cortical interneurons [63]. However, other studies [64–66] and only one meta-analysis [67] were unable to replicate this association.

Overall, even the most considered polymorphisms that may explain mechanisms of pharmacoresistant epilepsy, showed contradictory and inclusive results. Therefore, we assembled pharmacogenetics (PGt) and pharmacogenomics (PGx) studies reporting associations between AEDs resistant epilepsy and eventual polymorphisms. Then, we performed an updated meta-analysis to clarify their role in response to AEDs.

## Methods

We defined search strategy, study selection criteria, data elements and methods for study quality assessment.

### Data sources and literature searches

We conducted a literature search using Pubmed and Cochrane Library with English-language restriction from January 1980 to November 2016. The key words used in the search strategy were: “anti-epileptic drug(s)”, “antiepileptic drug(s)”, “anti epileptic drug(s)” and “epilepsy” and “efficacy”, “intractable”, “refractory”, “resistance”, “resistant”, “response to treatment”, “pharmacoresistance”, “pharmacoresistant” and “genetic factor(s)”, “genotype(s)”, “pharmacogenetic(s)”, “pharmacogenomic(s)”, “polymorphism(s)”, “variant(s)”, “variation(s)”, “SNP(s)”. We did not search of additional publications. The reported results followed the preferred reporting items for systematic reviews and meta-analyses guidelines (PRISMA).

### Eligibility and inclusion criteria

For eligibility, we retained full-text publications showing a relationship between genetic polymorphisms and responsiveness of AEDs in epilepsy (monotherapy or polytherapy).

The included studies met the following criteria: 1) Original research articles reported a genotypic evaluation of polymorphisms and resistant epilepsy to antiepileptic treatment. 2) Studies compared AEDs-resistant cases with AEDs-responsive cases. 3) Studies showed sufficient individual genotype frequencies for specific genotype model. 4) At least three studies on the same polymorphism were available in order to avoid the non-pertinence of the results and the high risk of bias.

### Data extraction

Two independent authors performed the data eligibility, they extracted the following information from each included study: first author, publication year, ethnicity of the study population, the number of cases and controls, genotype model for each polymorphism, age, gender, aetiology, type of epilepsy, and AEDs administered.

### Data synthesis and analysis

We calculated the association between polymorphisms and AEDs resistant epilepsy using individual and overall odds ratios (OR) with corresponding 95% confidence intervals (CIs) by Forest Plot (Comprehensive Meta-Analysis Version 3, USA). The *P*-value determined the significance of the combined ORs. If the *P*-value (*P*) < 0.05, we considered the pooled ORs statistically significant [68]. The *Z*-value showed uniformisation of values and their position in the full distribution of values in the program. The *I* ^2^ statistic test assessed statistical heterogeneity among included studies; if *I* ^2^ < 50%, fixed-effects model pooled study data and if *I* ^2^ ≥ 50%, random-effects model pooled it [69]. Additionally, we performed subgroup analysis using genotype model to quantify the reported association between polymorphisms and AEDs resistant epilepsy in each reported genotypic model. To identify publication bias between the included studies, we applied Funnel plot and Egger’s regression tests. The graph of Funnel plot reflected publication bias. Egger’s test assessed and confirmed funnel plot’s results: *P* < 0.05 determined the existence of bias [70].

## Results

### Evidence base

**Fig. 1:**
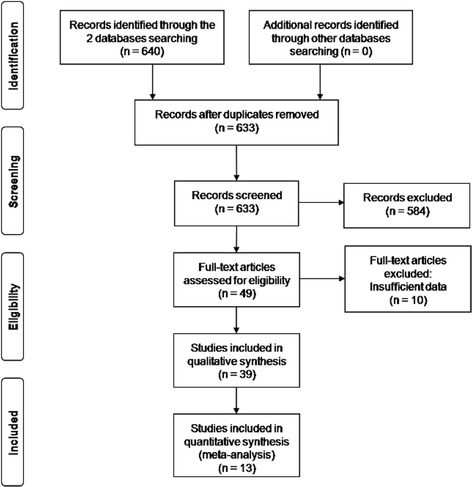
PRISMA flow diagram: study methodology of excluded and included articles

We identified a total of 640 potentially relevant articles. We excluded a total of 591 publications from the further analysis: abstract, articles showing absence of associations between polymorphisms and AEDs resistant epilepsy for insufficient data, case reports, duplicated articles, letter to the editors, meta-analysis, not epileptic studies, not human reports, researches about other treatments than AEDs, review articles and studies not related to associations between polymorphisms and AEDs resistant epilepsy (Fig. 1).

Among the 49 reports that met eligibility requirements], 39 reviewed an association between polymorphisms and epilepsy drug resistance [22–37, 50, 51, 62, 71–90]. We identified the majority of polymorphisms in AEDs transporter genes: *ABCB1* and *ABCC2*. We also found other polymorphisms in AEDs target genes: gamma-aminobutyric acid-a receptor alpha1-subunit (*GABRA1*), gamma-aminobutyric acid-a receptor alpha2-subunit (*GABRA2*), gamma-aminobutyric acid-a receptor alpha3-subunit (*GABRA3*), sodium channel nav1.1 (*SCN1A*), sodium channel nav1.2 (*SCN2A*), in other potential genes as apolipoprotein e (*ApoE*), cytochrome p450 1a1 (*CYP1A1*), cytochrome p450 family member 2c9 (*CYP2C9*), gamma-aminobutyric acid transporter 3 (*GAT3*), glutathione s-transferases mu 1 (*GSTM1*) and solute ligand carrier family 6 member a4 (*SLC6A4*). We summarized the characteristics of polymorphisms implicated in AEDs resistance in different ethnic groups (Table 1). We excluded 10 full-text studies for insufficient data (Fig. 1). Only 13 met the inclusion criteria and constituted the data set for this analysis [22–29, 31, 33–36] (Table 2).

**Table 1:** Characteristics of reviewed studies reporting associations between polymorphisms and AEDs resistance epilepsy

Gene	Polymorphism	Genotype model	Ethnicity	Reference
*ABCB1*	c.1199G > A (rs2229109)	GA vs. GG	Mexican	Escalante-Santiago et al. 2014 [71]
	c.1236T > C (rs1128503)	CC + CT vs. TT	Iranian	Maleki et al. 2010 [72]
	c.2677G > T/A (rs2032582)	AT + AG vs. GG + GT + TT	Mexican	Escalante-Santiago et al. 2014 [71]
		TT vs. GG + GT	European	Sánchez et al. 2010 [31]
			Malaysian	Subenthiran et al. 2013 [37]
		TT vs. GG		Subenthiran et al. 2013 [73]
			Japanese	Seo et al. 2006 [36]
	c.3435C > T (rs1045642)	CC vs. TT	Chinese	Hung et al. 2005 [22]
				Hung et al. 2007 [23]
			Egyptian	Ebid et al. 2007 [24]
			European	Sánchez et al. 2010 [31]
				Siddiqui et al. 2003 [25]
				Stasiołek et al. 2016 [26]
			Indian	Taur et al. 2014 [27]
			Iranian	Sayyah et al. 2011 [28]
			Thai	Keangpraphun et al. 2015 [29]
		CC vs. CT + TT	European	Basic et al. 2008 [30]
				Sánchez et al. 2010 [31]
		CC + CT vs. TT		Soranzo et al. 2004 [32]
		CT vs. CC + TT	Iranian	Sayyah et al. 2011 [28]
		TT vs. CC	Australian	Tan et al. 2004 [33]
			Chinese	Kwan et al. 2007 [34]
			Indian	Shaheen et al. 2014 [35]
			Japanese	Seo et al. 2006 [36]
		TT vs. CT + CC	Malaysian	Subenthiran et al. 2013 [37]
*ABCC2*	c.-24C > T (rs717620)	CT + TT vs. CC	Chinese	Qu et al. 2012 [74]
	c.-1019A > G (rs2804402)	AA vs. AG + GG	Indian	Grover et al. 2012 [75]
	c.-1549G > A (rs1885301)	GG vs. GA + AA		
	c.1249G > A (rs2273697)	AA vs. GG	Malaysian	Sha’ari et al. 2014 [50]
			Japanese	
			Chinese	
		GA vs. GG		
		GA + AA vs. GG		
		GA vs. GG + AA		Ma et al. 2014 [51]
	c.3972C > T (rs3740066)	CT vs. CC CC + TT vs. CC	Malaysian	Sha’ari et al. 2014 [50]
			Chinese	
		CT + TT vs. CC		Qu et al. 2012 [74]
		TT vs. CC + CT	Mexican	Escalante-Santiago et al. 2014 [71]
*ApoE*	c.388T > C (rs429358), c.526C > T (rs7412)	e3/4 vs. e3/3 + e2/3	European	Sporiš et al. 2005 [76]
	c.388T > C (rs429358)	e4 vs. e2 + e3	Chinese	Gong et al. 2016 [77]
*CYP1A1*	IVS1 + 606C > A (rs2606345)	CC + CA vs. AA CC vs. CA + AA	Indian	Grover et al. 2010 [78]
*CYP2C9*	c.1075A > C (rs1057910)	CYP2C9*3/*3 vs. CYP2C9*1/*1+ CYP2C9*1/*3	European	Seven et al. 2014 [79]
*GABRA1*	IVS11 + 15A > G (rs2279020)	GG vs. AA + AG	Indian	Kumari et al. 2010 [80]
				Kumari et al. 2011 [81]
	c.74 + 448C > T (rs6883877)	CC vs. TC + TT	Thai	Hung et al. 2013 [82]
*GABRA2*	g.46240004A > G (rs511310)	GG vs. AA + AG		
*GABRA3*	c.-27 + 37622A > G (rs4828696)	TT vs. CC + CT		
*GAT3*	c.1572C > T (rs2272400)	CT + TT vs. CC	Korean	Kim et al. 2011 [83]
*GSTM1*	GSTM1*0	GSTM1- vs. GSTM1+	Chinese	Liu et al. 2002 [84]
*SCN1A*	c.3184A > G (rs2298771)	AA vs. AG + GG		Wang et al. 2014 [85]
		AG + GG vs. AA		Zhou et al. 2012 [86]
		AG vs. AA + GG	Egyptian	Abo El Fotoh et al. 2016 [87]
	IVS5-91G > A (rs3812718)	AA vs. AG + GG	Japanese	Ma et al. 2014 [51]
				Abe et al. 2008 [62]
*SCN2A*	IVS7-32A > G (rs2304016)	AA vs. AG + GG	Chinese	Kwan et al. 2008 [88]
*SLC6A4*	5-HTTLPR	L/L vs. S/L + S/S	European	Hecimovic et al. 2010 [89]
	STin2 VNTR	12/12 vs. 10/10	Argentinean	Kauffman et al. 2009 [90]
		12/12 vs. 10/12 + 10/10	European	Hecimovic et al. 2010 [89]

*Abbreviation: ABCB1* atp-binding cassette sub-family b member 1, *ABCC2* atp-binding cassette subfamily c member 2, *ApoE* apolipoprotein e, *CYP1A1* cytochrome p450 1a1, *CYP2C9* cytochrome p450 family member 2c9, *GABRA1* gamma-aminobutyric acid-a receptor alpha1-subunit, *GABRA2* gamma-aminobutyric acid-a receptor alpha2-subunit, *GABRA3* gamma-aminobutyric acid-a receptor alpha3-subunit, *GAT3* gamma-aminobutyric acid transporter 3, *GSTM1* glutathione s-transferases mu 1, *SCN1A* sodium channel nav1.1, *SCN2A* sodium channel nav1.2, *SLC6A4* solute ligand carrier family 6 member a4

**Table 2:** Summary of studies included into meta-analysis

Polymorphism	Genotype Model	Ethnicity	Total No.		Male %/Female %	Mean Age (years)	Aetiology of epilepsy	Type of epilepsy	AEDs	Reference
*ABCB1* c.3435C>T	CC vs. TT	Chinese	Cases	331	56.193/43.807	39.1±11 ^a^	Cryptogenic	Generalized, partial	–	Hung et al., 2005 [22]
						38.5±13.4 ^b^	Cryptogenic, idiopathic			
			Controls	–	–	–	–	–	–	
			Cases	331	–	40.11±11 ^a^	Cryptogenic	Generalized, partial	CBZ, CNZ, GBP, LTG, OXC, PB, PHT, TPM, VGB, VPA	Hung et al., 2007 [23]
						39.5±13.4 ^b^	Cryptogenic, idiopathic			
			Controls	287	–	41±10.9	–	–	–	
		Egyptian	Cases	100	56 /44	35.9 ±8.42	–	Generalized, partial	PHT ^a^	Ebid et al., 2007 [24]
			Controls	50	64/36	38.6±10.32	–	–	–	
		European	Cases	289	49.827/50.173	27.0 ±18.5 ^a^ 26.0 ±19.8 ^b^	Various ^d^	Generalized, partial	–	Sánchez et al., 2010 [31]
			Controls	–	–	–	–	–	–	
			Cases	315	–	–	–	Generalized, partial	–	Siddiqui et al., 2003 [25]
			Controls	200	–	–	–	–	–	
			Cases	173	50.289/49.711	8.5±4.84 ^a^ 8.2±4.019 ^b^	–	–	CBZ, GBP, LEV, LTG, OXC, TPM	Stasiołek et al., 2016 [26]
			Controls	98	53.061/46.939	8.3±4.64	–	–	–	
		Indian	Cases	115	73.215/26.786	34.69±10.06 ^a^ 38.02±11.46 ^b^	–	–	CBZ, PB, PHT	Taur et al., 2014 [27]
			Controls	–	–	–	–	–	–	
		Iranian	Cases	332	52.711/47.289	28.8±11 ^a^ 27±13 ^b^	Various ^d^	Generalized, partial	CBZ, CNZ, LEV, LTG, OXC, PB, PHT, PRI, TPM, VPA	Sayyah et al., 2011 [28]
			Controls	–	–	–	–	–	–	
		Thai	Cases	110	52.727/47.273	41.96 ±12.19 ^a^ 46.65±12.65 ^b^	–	Generalized, partial	CBZ, PB, PHT, VPA	Keangpraphun et al., 2015 [29]
			Controls	–	–	–	–	–	–	
*ABCB1* c.3435C>T	TT vs. CC	Australian	Cases	609	–	–	–	Generalized, partial	–	Tan et al., 2004 [33]
			Controls	–	–	–	–	–	–	
		Chinese	Cases	746	–	–	Various ^d^	–	–	Kwan et al., 2007 [34]
			Controls	–	–	–	–	–	–	
		Indian	Cases	220	65.455/34.545	8.1±2.47 ^e^ 38.3±12.2 ^f^	Various ^d^	Generalized, partial	CBZ, CLB, LEV, OXC, PHT, VPA	Shaheen et al., 2013 [35]
			Controls	220	65.455/34.545	10.5±4.5 ^e^ 37±10 ^f^	–	–	–	
		Japanese	Cases	210	56.667/43.333	18.0±9.6 ^a^ 16.5±9.5 ^b^	Various ^d^	Generalized, partial	AZA, CBZ, CLB, CNZ, DZP, ESM, Ethotoin, NTZ, PB, PHT, VPA, ZNS	Seo et al., 2006 [36]
			Controls	–	–	–	–	–	–	

*Abbreviation*: *AEDs* anti-epileptic drugs, *ABCB1* atp-binding cassette sub-family b member 1, *AZA* acetazolamide, *CBZ* carbamazepine, *CLB* clobazam, *CNZ* clonazepam, *DZP* diazepam, *ESM* ethosuximide, *GBP* gabapentin, *LEV* levetiracetam, *LTG* lamotrigine, *NTZ* nitrozepam, *OXC* oxcarbazepine, *PB* phenobarbital, *PHT* phenytoin, *PRI* primidone, *TPM* topiramate, *VGB* vigabatrin, *VPA* valproate, *ZNS* zonisamide, − = no data, ^a^ AEDs-resistant cases, ^b^ AEDs-responsive cases, ^c^ Administration of PHT as monotherapy or polytherapy was not mentioned, ^d^ Idiopathic, cryptogenic, symptomatic, ^e^ <15 years, ^f^ >15 years

### Data analysis

We carried out a meta-analysis to evaluate the relationship between *ABCB1* C3435T polymorphism and AEDs resistance among AEDs-resistant patients vs. AEDs-responsive patients. The included studies were heterogeneous for the study characteristics. The analysis of data showed that 454 of 1653 AEDs-resistant patients (27.465%) and 282 of 1732 AEDs-responsive patients (16.282%) were included in the statistical analysis [22–29, 31, 33–36]. The frequency of AEDs-resistant cases was higher than AEDs-responsive patients. We divided the age of cases and controls into three subgroups: >20 years, 20–40 years, and <40 years. We divided the gender of cases and controls into two subgroups: males >50% and males <50%. A total of eight included studies were conducted in Asia [22, 23, 27–29, 34–36], three studies in Europe [25, 26, 31], one study in Egypt [24] and one another in Australia [33]. We classified the cases by epilepsy syndrome (idiopathic, cryptogenic or symptomatic epilepsy) [22, 23, 28, 31, 34, 36] or by seizure types (generalized or partial seizures) [22–24, 28, 29, 31, 33, 35, 36]. However, the classifications of cases by epilepsy syndrome were not mentioned in seven studies [24–27, 29, 33, 35] and the classifications of cases by seizure types were not mentioned in three studies [26, 27, 34]. Two studies were stratified by epilepsy syndrome [28, 31] and three studies were stratified by seizure types [29, 33, 35]. Cases were treated with AEDs polytherapy in seven studies [23, 26–29, 35, 36]. Only one study reported association between *ABCB1* C3435T polymorphism and cases with Phenytoin (PHT) therapy, the administration of PHT as monotherapy or polytherapy was not mentioned [24]. However, AEDs were not specified in five studies [22, 25, 27, 31, 33]. We summarized the characteristics of the available included studies in Table 2.

### Association of *ABCB1* C3435T polymorphism with the susceptibility to AEDs resistance

**Fig. 2:**
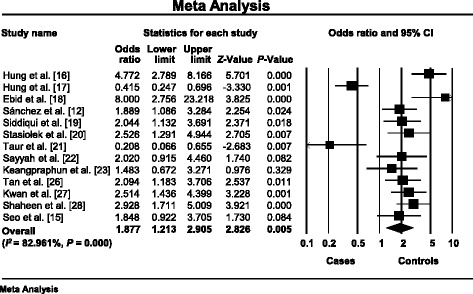
Association between *ABCB1* C3435T polymorphism and AEDs resistant epilepsy. Forest plot showed individual and overall ORs (black squares) with corresponding 95% CIs (horizontal bars) by individual report. *P*-value showed statistical significance of ORs and *Z*-value showed uniformisation of values and its position in the full distribution of values. Heterogeneity between the studies was mentioned

The heterogeneity among the included studies was high (*I* ^2^ = 82.961%, *P* < 10^-3^) and we used a random-effects model [22–29, 31, 33–36]. The summary OR was 1.877, 95% CI 1.213–2.905, *P* = 0.005 showing that *ABCB1* C3435T was significantly associated with AEDs resistance (Fig. 2).

**Fig. 3:**
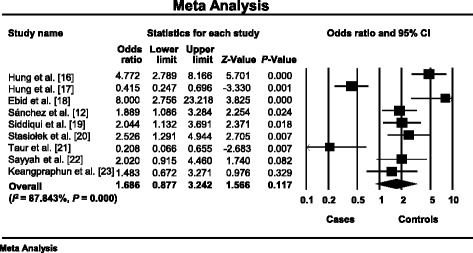
Association between *ABCB1* 3435CC genotype and AEDs resistant epilepsy. Forest plot showed individual and overall ORs (black squares) with corresponding 95% CIs (horizontal bars) by individual report. *P*-value showed statistical significance of ORs and *Z*-value showed uniformisation of values and its position in the full distribution of values. Heterogeneity between the studies was mentioned

**Fig. 4:**
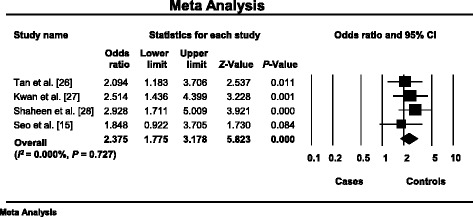
Association between *ABCB1* 3435TT genotype and AEDs resistant epilepsy. Forest plot showed individual and overall ORs (black squares) with corresponding 95% CIs (horizontal bars) by individual report. *P*-value showed statistical significance of ORs and *Z*-value showed uniformisation of values and its position in the full distribution of values. Heterogeneity between the studies was mentioned

For the robustness of our findings, we used subanalysis by dominant (CC vs. TT) and recessive (TT vs. CC) genotype models. The heterogeneity among the nine included studies was high (*I* ^2^ = 87.843%, *P* < 10^-3^) in the dominant model [22–29, 31]. The summary OR was 1.686, 95% CI 0.877–3.242, *P* = 0.117 under a random-effects model (Fig. 3). The analysis of the recessive model revealed that the heterogeneity was absent (*I* ^2^ = 0.000%, *P* = 0.727) among the four included studies [33–36]. The summary OR was 2.375, 95% CI 1.775–3178, *P* < 10^-3^ under a fixed-effects model (Fig. 4). Therefore, the results of our present meta-analysis indicates that the association of *ABCB1* C3435T polymorphism with the risk of AEDs resistance, exists and it is more significant in *ABCB1* 3435TT genotype than in 3435CC genotype.

### Analysis of publication bias

**Fig. 5:**
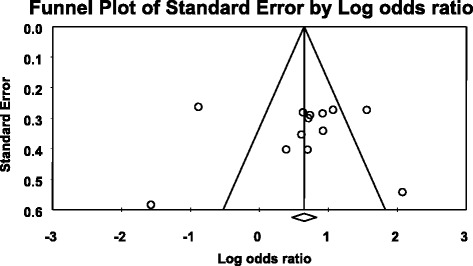
Publication bias of the association between *ABCB1* C3435T polymorphism and AEDs resistant epilepsy

**Fig. 6:**
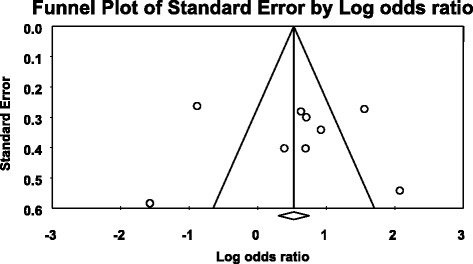
Publication bias of the association between *ABCB1* 3435CC genotype model and AEDs resistant epilepsy

**Fig. 7:**
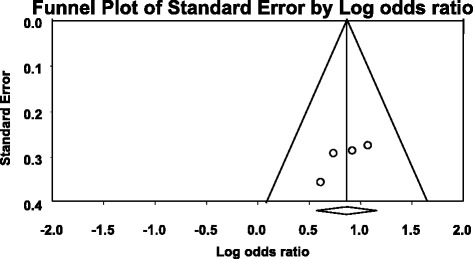
Publication bias of the association between *ABCB1* 3435TT genotype model and AEDs resistant epilepsy

For the association between *ABCB1* C3435T polymorphism, *ABCB1* 3435CC, and 3435TT genotype models with AEDs resistance, Funnel Plot showed asymmetrical appearances (Figs. 5, 6 and 7) and Egger’s regression test showed that *P* = 0.413, *P* = 0.492, and *P* = 0.085, respectively, were more than 0.05. The two tests demonstrated a significant publication bias.

## Discussion

Epilepsy is a serious health problem affecting about 65 million people worldwide and manifesting many syndromes and types of seizures [60]. Since uncontrollable seizures increase morbidity and mortality, drug-resistant epilepsy is one of the major problems that physicians encounter. Recurrent seizures can devastate patients and their families. Therefore, drug-resistant epilepsy still remains one of the main challenges for epileptologists.

Since that genetic polymorphisms may play a role in response to AEDs [10], we conducted an updated systematic review in order to summarize the impact of polymorphisms in *ABCB1*, *ABCC2*, *ApoE*, *CYP1A1*, *CYP2C9*, *GABRA1*, *GABRA2*, *GABRA3*, *GAT3*, *GSTM1*, *SCN1A*, *SCN2A*, and *SLC6A4* genes on AEDs resistant epilepsy. Our meta-analysis concerned only the association between *ABCB1* C3435T polymorphism and drug-resistant epilepsy, which revealed a significant risk to pharmacoresistance (OR = 1.877, 95% CI 1.213–2.905, *P* = 0.005) (Fig. 2). Some studies confirmed our results [22–37]. Nevertheless, many other reports failed to prove an association between *ABCB1* C3435T polymorphism and refractory epilepsy [38–42, 91–96].

The first publication showed that drug-resistant patients compared to drug-responsive patients, were more likely to have the CC genotype than the TT genotype (*P* = 0.006) [25]. Zimprich et al. confirmed the result [97]. Moreover, many studies indicated that the CC genotype were more prevalent in drug-resistant epilepsy [12, 16–23]. However, three Asian studies [34–36] and one Australian study [33] showed the opposite association of TT genotype high frequency. In addition, our meta-analysis showed that patients resistant to AEDs were more likely to have *ABCB1* 3435TT genotype (OR = 2.375, 95% CI 1.775–3.178, *P* < 10^-3^) than 3435CC genotype (OR = 1.686, 95% CI 0.877–3.242, *P* = 0.117) (Figs. 3 and 4).

Due to these controversial results, meta-analyses were made in order to clarify the association between *ABCB1* C3435T polymorphism and drug-resistant epilepsy. The majority suggest that the *ABCB1* C3435T polymorphism may not be involved in the response to AEDs [58–62]. The study of Bournissen et al. showed no association of *ABCB1* C3435T polymorphism with risk of drug resistance in overall and in the subgroup analysis by ethnicity (Asian and Caucasian populations) (*n* = 3371 subjects) [43]. The first study of Haerian et al. demonstrated the lack of allelic association with the risk of drug resistance under fixed and random effects models (*n* = 6755 subjects) [44] and the second study of Haerian et al. showed no significant association of *ABCB1* alleles, genotypes, and haplotypes with recurrent seizures (*n* = 7067 patients) [45]. In the two studies, subanalysis of studies by ethnicity (Asian and Caucasian populations) yielded similar findings. Nurmohamed et al. failed to find a statistical significance between genotypes of *ABCB1* C3435T polymorphism in cases and controls (*n* = 3996 subjects) [46]. No allelic neither genotypic association of *ABCB1* C3435T polymorphism with childhood risk of drug resistance was found in overall and in the subgroup analysis by ethnicity (Asian and Caucasian populations) (*n* = 1249 subjects) in the study of Sun et al. [47]. Recently, two meta-analyses have indicated that CC genotype was associated with recurrent seizures in Caucasians. However, none of the genetic comparisons exhibited a significant association in Asians [63, 64]. In our knowledge, no another meta-analysis showed the same result as ours. Overall, meta-analyses stratified by genotype genetic models in the overall studies, indicate that the polymorphism may not play a major role in drug resistance to AEDs [46] and similar results are found in the subgroup analysis for the Asian and the Caucasian populations [43–45, 47]. However, other meta-analyses show a significant association in a specific ethnic subgroup [63, 64]. These discrepant results are mainly due to the small sample size, which is a common problem in association studies leading to underpowered genotypic results. Worldwide collaboration between different centers is then necessary to increase the sample size. In addition, ethnicity is another factor that may affect the results. An allele may become more common in ethnic subgroup but not in another, which may affect the response to AEDs [45]. However, four meta-analyses show no evidence that the *ABCB1* C3435T polymorphism is associated with the risk of resistance to AEDs in Asians and Caucasians [43–45, 47]. Therefore, meta-analysis startified by ethnicity are needed to increase in order to confirm the ethnic-dependence of AEDs resistant epilepsy.

AEDs transporters have contribute in pharmacoresistant epilepsy. In fact, the most studied AEDs transporter proteins like membrane proteins, are ABC transporter superfamily members. They are ATP-dependent drug efflux pumps for specific AED and are mainly encoded by *ABCB1* gene. ABCB1 protein or P-gp was transporte AED in the BBB [72]. P-gp activity can be affected by *ABCB1* polymorphisms reducing plasmatic levels of AEDs and minimizing antiepileptic treatment efficiency in epileptic patients [98, 99]. If genetic background affects the expression of P-gp, then penetration of AEDs in the brain might depend on the patient’s genotype [16, 18]. Homozygous TT genotype is associated with decreased P-gp expression [4, 100].

Compared to literature search supporting conflicting results, our results show a higher contribution of *ABCB1* 3435TT genotype on response to AEDs. Our findings may contribute to exhibit the implication of genetic markers in refractory epilepsy before starting the treatment. In order to have a better AEDs therapeutic response, the identification of new potential genetic markers become necessary against pharmcoresistance in epilepsy. This will lead to a better understanding of drug resistance mechanisms in epilepsy. Furthermore, it will be extremely important for individual AEDs selection, early surgery feasibility and development of new efficacious treatments.

### Limitations

Our analysis is consistent to our strategy search, inclusion criteria and statistical parameters. However, it may be limited due to several factors: 1) Few number of included studies is insufficient to carry out a subgroup analysis by ethnicity. In addition, the ethnicities in the included studies are heterogeneous. PGt and PGx studies of AEDs resistance should be performed by ethnicity. 2) Publication bias and heterogeneity might have an impact on the meta-analysis results. 3) Most of the included studies match different types of epilepsy with different AEDs. The affinity of each AED for ABC transporters is variable. In fact, Valproic acid (VPA) is a widely used AED and it is not transported by P-gp [101]. Thereby, the association between *ABCB1* C3435T polymorphism and drug resistance epilepsy could be affected. Correlation between PGt and PGx results with specific AED should be required. 4) Different inclusion criteria are used to classify AEDs-resistant patients in the included studies, subsequently, the interpretation of the meta-analysis results become very complex. In fact, AEDs-resistant patients were defined as patients who had at least one seizure per month or 10 seizures over the previous year, despite two or more AEDs at therapeutic dosages and/or serum drug concentrations in three studies [22, 28, 34]. In other reports, drug resistance was defined as the occurrence of at least four seizures over the year despite more than three appropriate and tolerated AEDs for the epilepsy syndrome [25, 31, 33]. In some studies, it was defined as the failure of two appropriate and tolerated AEDs trials [27, 29], with a poor clinical outcome and recurrent seizures [35], or the occurrence of any types of seizures for a minimum of one year at the same dose of AEDs [36], or any seizures during the past three months [24] and more than 10 seizures over the year [23].

## Conclusions

Various studies have yielded contradictory findings regarding the relationship between *ABCB1* C3435T polymorphism and AEDs resistance in epilepsy. In the current meta-analysis, we demonstrate the existence of a statistical significant association between *ABCB1* 3435TT genotype and refractory epilepsy. Therefore, the screening of *ABCB1* gene for this polymorphism in the future might be useful to decide the best treatment option for each patient and to predict the treatment outcome for new epileptic patients. However, considering the few number of included studies and the significant publication bias found in this meta-analysis, further investigations should be helpful to validate the use of this polymorphism in treatment decisions.

## Abbreviations

ABC: atp-binding cassette
*ABCB1*: atp-binding cassette sub-family b member 1
*ABCC2*: atp-binding cassette sub-family c member 2
AEDs: Antiepileptic drugs
*ApoE*: Apolipoprotein e
BBB: Blood–brain barrier
CBZ: Carbamazepine
CIs: Confidence intervals
*CYP1A1*: Cytochrome p450 1a1
*CYP2C9*: Cytochrome p450 family member 2c9
*GABRA1*: Gamma-aminobutyric acid-a receptor alpha1-subunit
*GABRA2*: Gamma-aminobutyric acid-a receptor alpha2-subunit
*GABRA3*: Gamma-aminobutyric acid-a receptor alpha3-subunit
*GAT3*: Gamma-aminobutyric acid transporter 3
*GSTM1*: Glutathione s-transferases mu 1
ILAE: International league against epilepsy
*MDR*1: Multidrug resistance protein 1
*MDR2*: Multidrug resistance protein 2
ORs: Odds ratios
OXC: Oxcarbazepine
*P*: *P*-value
P-gp: P-glycoprotein
PGt: Pharmacogenetics
PGx: Pharmacogenomics
PHT: Phenytoin
PRISMA: Preferred reporting items for systematic reviews and meta-analyses guidelines
*SCN1A*: Sodium channel nav1.1
*SCN2A*: Sodium channel nav1.2
*SLC6A4*: Solute ligand carrier family 6 member a4
SNP: Single nucleotide polymorphism
VPA: Valproic acid
